# Inhibition of histone deacetylases attenuates tumor progression and improves immunotherapy in breast cancer

**DOI:** 10.3389/fimmu.2023.1164514

**Published:** 2023-03-09

**Authors:** Bi Lian, Xiaosong Chen, Kunwei Shen

**Affiliations:** Department of General Surgery, Comprehensive Breast Health Center, Ruijin Hospital, Shanghai Jiao Tong University School of Medicine, Shanghai, China

**Keywords:** HDAC, inhibitors, breast cancer, immunotherapy, targets

## Abstract

Breast cancer is one of the common malignancies with poor prognosis worldwide. The treatment of breast cancer patients includes surgery, radiation, hormone therapy, chemotherapy, targeted drug therapy and immunotherapy. In recent years, immunotherapy has potentiated the survival of certain breast cancer patients; however, primary resistance or acquired resistance attenuate the therapeutic outcomes. Histone acetyltransferases induce histone acetylation on lysine residues, which can be reversed by histone deacetylases (HDACs). Dysregulation of HDACs *via* mutation and abnormal expression contributes to tumorigenesis and tumor progression. Numerous HDAC inhibitors have been developed and exhibited the potent anti-tumor activity in a variety of cancers, including breast cancer. HDAC inhibitors ameliorated immunotherapeutic efficacy in cancer patients. In this review, we discuss the anti-tumor activity of HDAC inhibitors in breast cancer, including dacinostat, belinostat, abexinostat, mocetinotat, panobinostat, romidepsin, entinostat, vorinostat, pracinostat, tubastatin A, trichostatin A, and tucidinostat. Moreover, we uncover the mechanisms of HDAC inhibitors in improving immunotherapy in breast cancer. Furthermore, we highlight that HDAC inhibitors might be potent agents to potentiate immunotherapy in breast cancer.

## Introduction

Breast cancer is one of the common tumors worldwide. Approximately 2.3 million new breast cancer cases were estimated in 2020 in the 185 countries (1). It has been estimated that there are 297,790 new cases of breast cancer and 59,910 deaths due to this deadly disease in the United States (2). Approximately 11%-20% of breast cancer patients are triple negative breast cancer (TNBC) due to lack of expression of HER2, ER and PR (3). TNBC patients often have aggressive behavior, metastasis and poor prognosis (4). For the treatment of local breast cancer, there are surgery and radiation, while the systemic therapies of breast cancer include chemotherapy, hormone therapy, targeted drug therapy and immunotherapy (5, 6). Histone acetyltransferases can lead to histone acetylation on lysine residues, which can be reversed by histone deacetylases (HDACs) (7, 8). It has been known that HDACs function on remodeling of chromatin and modulation of gene expression by specific epigenetic regulation (9). There are 18 HDACs that have been characterized to regulate various biological processes, which are classified into four groups (I-IV). Class I includes HDAC1, HDAC2, HDAC3 and HDAC8, which are related to RPD3 gene. Class II includes HDAC4, HDAC5, HDAC6, HDAC-7, HDAC9 and HDAC10. Class III includes sirtulin 1-7 and class IV includes HDAC11 (10–12). Dysregulation of HDACs *via* mutation and abnormal expression contributes to oncogenesis and tumor progression (10–12). Therefore, modulation of HDACs could be a potent strategy for cancer treatment.

## Role of HDAC in immunotherapy

Immunotherapy has emerged for fighting cancer *via* using the patient’s own immune system (13). Immunotherapy includes monoclonal antibodies, chimeric antigen receptor (CAR) T-cell therapy, CAR NK cell therapy, tumor infiltrating lymphocyte (TIL) therapy, endogenous T cell (ETC) therapy, immune checkpoint inhibitors (ICIs), cancer vaccines, cytokines and immunomodulators (14–17). It has been known that ICIs block immune checkpoints, which allow immune cells to respond to tumor. Inhibitory immune checkpoint molecules include programmed cell death ligand (PD-1), programmed death ligand (PD-L1), PD-L2, B7-H3 (CD276), B7-H4 (VTCN1), LAG3, TIM-3, and cytotoxic T-lymphocyte-associated antigen 4 (CTLA-4) (18, 19). Although immunotherapy has improved the survival of certain cancer patients, primary resistance and acquired resistance in immunotherapy attenuate the cancer treatment outcomes (20, 21). Hence, it is pivotal to uncover the mechanism of immunotherapy resistance and to develop the compounds that improve immunotherapy.

Several HDAC inhibitors have been developed and exhibited the potent anti-tumor activity in a various cancer types, including inhibition of tumor growth, metastasis and drug resistance (22–24). For instance, abexinostat, givinostat and mocetinostat decreased the expression of Slug and increased the expression of E-cadherin in mammary tumor cells (25). Breast epithelial cells with E-cadherin depletion were sensitive to several HDAC inhibitors, including entinostat, vorinostat, pracinostat, and mocetinostat, due to inhibition of proliferation and upregulation of cell apoptosis (26). Here, we discuss the function of HDAC inhibitors in tumorigenesis, especially in improving immunotherapy in breast cancer.

### Vorinostat

Vorinostat, also known as SAHA (suberoylanilide hydroxamic acid), is an oral inhibitor of class I and II of HDACs, which was the first time to approve for clinical application in patients with cutaneous T-cell lymphoma in 2006 (27–29). Vorinostat has been determined by preclinical experiments and clinical trials to decide its therapeutic efficacy in combination with other antitumor drugs in breast cancer (30). Vorinostat plus CDK inhibitor flavopiridol treatments exhibited synergistic lethality in breast cancer cells *via* suppression of ERK1/2 and AKT pathways and regulation of apoptosis pathways (31). Using breast cancer brain metastatic cells and intracranial xenograft model, radio-sensitivity was increased by vorinostat (32). Vorinostat accelerated radio-sensitivity of breast tumor cells, leading to suppression of lung metastasis *via* inhibition of MMP-9, DNA repair proteins and modulation of autophagy and endoplasmic reticulum stress (33).

TRAIL-resistant breast cancer cells became more sensitive after vorinostat treatment in BALB/c nude mice because vorinostat inhibited the expression of NF-κB, cyclin D1, Bcl-2, Bcl-xL, VEGF, MMP-2, MMP-9, HIF-1α, IL-6, IL-8, increased the expression of DR4, DR5, p21, PUMA, TIMP-1, TIMP-2, Bax, Bak, Bim and Noxa (34). It has been reported that vorinostat overcame apoptosis-inducing ligand Apo2L/TRAIL resistance *via* regulation of Bax, DR5, caspase-3, caspase-8, caspase-9 and PARP cleavage in human MDA-MB-231 breast cancer cells (35). Vorinostat increased the sensitivity of olaparib, one PARP inhibitor, in TNBC cells *via* induction of DNA damage, apoptosis and autophagy (36). Vorinostat restrained brain metastasis and stimulated DNA double-strand breaks and induced the downregulation of Rad52 in a TNBC model (37). Vorinostat promoted taxol-mediated cell death and triggered inhibition of cell growth and induced cell cycle arrest at G2/M phase in breast cancer (38). Vorinostat in combination with Aurora kinase inhibitor (MK-0457) displayed synergistical inhibition of proliferation of breast cancer cells (39). Vorinostat activated the expression of estrogen receptor α (ERα) and sensitized a ligand of the aryl hydrocarbon receptor, aminoflavone, -mediated growth inhibition in mesenchymal-like TNBC cells, such as MDA-MB-231 and Hs578T cells (40). Co-treatment with vorinostat and simvastatin exhibited synergistic functions on cell proliferation and apoptosis *via* inhibition of Rab7 prenylation in TNBC cells (41). It has been found that tamoxifen sensitivity was enhanced by vorinostat treatment in TNBC cells (42).

Vorinostat in combination with chemotherapeutic agent decitabine increased sensitivity of Fas ligand (FasL)-induced apoptosis and CTL immunotherapy *via* promotion of CD8+ T cells in colon cancer cells (43). Vorinostat increased sensitivity of anti-GD2 monoclonal antibody (mAb) treatment and reduced tumor growth through elevation of macrophage effector cells with high expression of Fc-receptors and reduction of MDSC number in neuroblastoma (44, 45). In pancreatic cancer, vorinostat and sorafenib co-treatment enlarged the efficacy of anti-PD-1 antibody *via* promotion of CD8+ cells, M1 macrophages and NK cells in mice (46). A combination therapy by vorinostat and anti-PD-L1 to abrogate the immune escape has been reported *via* induction of cell apoptosis and G1 phase arrest in melanoma (47). In head and neck and salivary cancer patients with vorinostat plus pembrolizumab treatments, NLR, neutrophils, lymphocytes and T helper cells were associated with poor overall survival (48). The MDA-MB-231 breast carcinoma cells and LNCaP prostate cancer cells displayed sensitivity to vorinostat therapy *via* enhancement of the immune evasion, leading to promotion of T-cell-induced lysis. HDAC1 was further identified to play a pivotal role in tumor immune escape in breast cancer cells (49). Data from ER-positive breast cancer patients after vorinostat, tamoxifen and pembrolizumab treatments revealed that exhausted T cell signature was linked to immunotherapy response (50). Hence, combination of HDAC inhibitors and immunotherapy could obtain synergistic effects in cancer therapy in breast cancer.

### Entinostat

Entinostat, a class I HDACs inhibitor, has been uncovered to attenuate cell proliferation and stimulated cell apoptosis in breast cancer (51, 52). Moreover, entinostat was critically involved in reversal of tumor immune escape in breast cancer (51). One study revealed that entinostat promoted lapatinib efficacy *via* inhibition of AKT phosphorylation, activation of FOXO3 transcription, leading to elevation of Bim1 expression in breast cancer cells with HER2 overexpression (53). Entinostat can attenuate the resistance of trastuzumab/lapatinib-resistant breast cancer cells with HER2 overexpression to the trastuzumab/lapatinib treatment (53). Entinostat plus MEK inhibitor pimasertib retarded cell growth in TNBC cells and inflammatory breast cancer (IBC) cells, and reduced tumor growth in mice *via* regulation of NOXA-participated MCL1 degradation (54).

One study used microarray analysis and revealed that doxorubicin and entinostat regulated numerous gene expressions related to differentiation, inflammation and proliferation. Entinostat sensitized doxorubicin-mediated cell cycle arrest at G2 phase (55). Doxorubicin and entinostat inhibited the expression of E2F and Myc genes, elevated interferon genes and increased the numbers of tumor-infiltrating lymphocytes. Moreover, entinostat and doxorubicin enhanced the expression of tumor testis antigens, such as IL13RA2, and elevated the expression of ICOSL and GITRL in MDA-MB-231 cells, which were immune checkpoint agonists (55). PD-L1 expression was increased by entinostat and reduced by doxorubicin treatment. Entinostat, all-trans retinoic acid, and doxorubicin together stimulated cell death and differentiation, leading to regression of tumor growth in mice by a xenograft model of TNBC (55). A combination of entinostat, all-trans retinoic acid, and doxorubicin caused tumor regression *via* targeting tumor-initiating cells in TNBC and modulating the ESE-1 and ELF-3 (56).

Entinostat, a cancer vaccine, and an IL15 agonist N-803 displayed a synergistic effect on tumor growth *via* upregulation of infiltration of CD8+ T cells, promotion of tumor inflammation-related gene expressions, enhancement of T cell responses to antigens, reduction of VISTA expression in 4T1 TNBC murine carcinoma model and MC38-CEA colon mouse model (57). Combined treatments with vaccine, entinostat, ICIs, and chemotherapy had exhibited a potential efficacy in advanced breast cancer (58). The breast cancer cells and prostate tumor cells exhibited sensitivity to entinostat by T-cell-involved lysis (49). Entinostat altered the tumor-related antigens, including PSA, brachyury, CEA and MUC1, and elevated the expression of several proteins that governed tumor immune recognition and antigen processing (49). Entinostat combined with immunotherapy could be a potential strategy for breast cancer therapy.

### Romidepsin

Romidepsin (FK228), a class I HDAC inhibitor, has been reported to inhibit the tumor growth in different types of cancers (59, 60). For example, in colon cancer cells, romidepsin attenuated cellular immune functions *via* upregulation of PD-L1 expression by enhancing the acetylation of histones H3 and H4 and modulation of BRD4 (61). Romidepsin accelerated the number of FOXP3+ regulatory T cells, reduced the number of IFN-γ+ CD8+ T cells, and alleviated Th1/Th2 ratio in TME in subcutaneous model and colitis-related cancer mice. Moreover, Romidepsin-mediated tumor suppression was abrogated by anti-PD-1 antibody treatment in colon cancer cells (61). One case report showed that romidepsin might be safe and effective for treatment of anaplastic large cell lymphoma (ALCL), which did not impair cellular immunity to HTLV-1 (62).

Romidepsin increased paclitaxel sensitivity and blocked tumor metastasis in inflammatory breast cancer (63). Specifically, romidepsin impaired tumor emboli and lymphatic vascular structure, and suppressed the expression of VEGF and HIF-1α in inflammatory breast cancer. Moreover, romidepsin induced the expression of acetylated Histone 3 proteins, triggered cell apoptosis and upregulated p21 expression level (63). Recently, romidepsin treatment upregulated the expression of chemokines, stimulated T-cell infiltration, and promoted T-cell-induced tumor regression. A combination of romidepsin and PD-1 blockade elevated T-cell infiltration and increased the efficacy of anti-PD-1 immunotherapy in lung adenocarcinoma (64). One group reported that a triple combination (gemcitabine, romidepsin, cisplatin) accelerated cell death in MDA-MB-231 and MDA-MB-468 cells (65). Moreover, a triple combination treatment using gemcitabine, romidepsin and cisplatin inhibited cell survival and invasion *via* targeting EMT in an ROS-dependent way, leading to suppression of tumor development, recurrence, and metastasis in TNBC (66).

### Panobinostat

It has been known that panobinostat (LBH589), a pan-HDAC inhibitor, performs a tumor suppressive function in various cancer types (67, 68). The function of panobinostat has bene verified in breast carcinogenesis and progression. Panobinostat enhanced the acetylation of GRP78 (glucose-regulated protein 78) and increased endoplasmic reticulum stress *via* upregulation of p-eIF2α, CHOP and ATF4, and elevation of BIK, BIM, Bax and BAK expression, acceleration of the caspase-7 activity and UPR in breast cancer cells (69). Panobinostat inhibited proliferation of breast cancer cells *via* modulation of aromatase gene expression, and synergized the anti-tumor function of letrozole in hormone-dependent breast cancer (70). In addition, panobinostat exposure elevated histone acetylation, induced G2/M cell cycle arrest and alleviated cell proliferation in TNBC cells. Panobinostat increased the expression of E-cadherin and changed the cell morphology in MDA-MB-231 cells (71). Another study showed that panobinostat inhibited the expression of ZEB family (ZEB1 and ZEB2) and led to suppression of tumor metastasis in TNBC (72).

The proliferation of breast cancer cells with aromatase inhibitor resistance was mitigated by panobinostat in part *via* inactivation of NF-κB1 pathway (73). The invasive and migratory ability of breast cancer cells was also repressed by panobinostat *via* induction of E-cadherin and alteration of Slug, MTA3 and Snail (74). Using a claudin-low TNBC PDX model, one group revealed that panobinostat inhibited the mesenchymal phenotype, such as inhibition of collagen expression (75). Panobinostat accelerated the expression of APCL and blocked Wnt/β-catenin pathway *via* promotion of β-catenin degradation in breast cancer, resulting in inactivation of β-catenin targets, including c-Myc, CD44, Cyclin D1 and c-Jun, which contributed to inhibition of tumor growth and metastasis (76). Panobinostat plus rapamycin led to increased efficacy against TNBC on inhibition of proliferation, invasion, migration and induction of apoptosis, which could be due to overproduction of ROS ad activation of endoplasmic reticulum stress in breast cancer (77). Panobinostat inhibits tumor growth by induction of autophagy and accelerated secretory autophagy *via* targeting Vps34/Rab5C pathway in breast cancer (78). Panobinostat has shown the treatment benefits in oncolytic herpes simplex virus in combination with anti-PD-1/PD-L1 therapy in glioma and squamous cell carcinoma (79). The efficacy of panobinostat was spatially correlated with multiple gene expressions, including galectin-3, cleaved caspase-3, PD-L1, neuropilin-1 and calrecticulin in breast cancer, suggesting that panobinostat (80). Without a doubt, the function of panobinostat in altering immunotherapy warrant to further exploration in breast cancer.

### Mocetinotat

Mocetinostat, a class I/IV HDAC inhibitor, has been identified to suppress the tumorigenesis and tumor development in a various types of human cancers (81). Mocetinostat increased PD-L1 expression and elevated the expression of antigen presentation genes in NSCLC (82). Mocetinostat interacted with the promoters of a class I HDAC and increased active histone marks, and enhanced IFN-γ activity in governing class II transactivator. In mice, mocetinostat reduced the number of Tregs and MDSCs, but elevated the number of CD8+ population in tumors. Mocetinostat and PD-L1 antibody displayed a synergistic function in mouse lung tumor models (82). Mocetinostat plus the BET inhibitor JQ1 reduced viability of breast cancer cells *via* modulation of cell cycle-associated gene expressions. Mocetinostat and JQ1 cotreatment upregulated the expression of USP17 family members in breast cancer cells, resulting in inactivation of Ras/MAPK pathway and attenuation of cell viability (83).

Fyn-related kinase (FRK) has been known to be repressed in cancer cells due to its promoter CpG methylation (84). Cell migration and invasion were reduced by FRK overexpression *via* inactivation of MAPK, AKT and JAK/STAT pathways and blockade of EMT in breast cancer cells, including inhibition of slug, vimentin, fibronectin, and upregulation of E-cadherin (85). Mocetinostat and entinostat can induce re-expression of FRK at mRNA and protein levels in basal B breast cancer cells, contributing to tumor regression (86). Similarly, mocetinostat exhibited anti-cancer functions in basal-like breast cancer cells with HDAC2 overexpression (87). Moreover, mocetinostat plus azacytidine increased chemotherapeutic sensitivity in mammary mesenchymal tumors *via* targeting EMT process (25). One group used TCGA database and found that mocetinostat and vorinostat exhibited the functional similarity with the FDA-approved drugs for the treatment of HER2-postive breast cancer (88). Mocetinostat combined with capecitabine showed a synergistic effect on suppression of proliferation and induction of apoptosis in 4T1 breast cancer cells *via* targeting Bax, Bcl-2, PI3K/AKT, c-Myc, PTEN, p53, caspase-7, -9, and cleaved PARP (89). It is required to further dissect the function of mocetinostat in improving immunotherapy in breast cancer.

### Abexinostat

Abexinostat (PCI-24781, CRA-024781) is a Pan-HDACs mainly targeting HDAC1. It has been reported that abexinostat increased tumor radio-sensitivity in NSCLC (90). PCI-24781 was developed to decrease cell proliferation, differentiation and metastasis *via* influencing calcium influx by activation of RGS2 in breast cancer (91). Abexinostat triggered the differentiation of cancer stem cells in breast cancer with low level of lncRNA Xist expression (92). Moreover, low expression of lncRNA Xist could indicate abexinostat response in breast tumor PDXs and linked to an inhibition of cancer stem cells in breast cancer (92). Interestingly, administration of abexinostat did not change the expression of ESR1, ERα, and ESR1-associated genes in xenograft models (93). This study indicated that it is doubtable to use a combination of abexinostat and hormone therapy for the management of breast cancer patients. Due to unclear role of abexinostat in immune response, it is pivotal to define the function of abexinostat in regulation of immunotherapy of breast cancer patients.

### Belinostat

Belinostat (Beleodaq, PXD101) is a HDACi with antineoplastic function in part *via* targeting HDAC6. One study showed that TNBC cells and HER2-enriched breast cancer cells were remarkably sensitive to belinostat and panobinostat treatment. Moreover, belinostat and panobinostat increased doxorubicin sensitivity in TNBC cells (94). Belinostat and SAHA sensitized TNBC cells to the PARP inhibitor olaparib treatment, showing the synergistic inhibition of proliferation of TNBC cells and induction of cell apoptosis (95). Belinostat plus Hsp90 inhibitor 17-AAG displayed a synergistic effect on suppression of invasion and cell growth in TNBC cells *via* inhibiting the expression of TEAD family proteins and elevating YY1AP1 phosphorylation and MLC1 (modulator of VRAC current 1) (96). Chemotherapeutic drugs led to cancer stem cell (ALDH+/CD44+) abundance in breast cancer, which was abrogated by belinostat exposure (97). One group has demonstrated that belinostat stimulated the expression of CXCL1 in TBNC cells, suggesting that CXCL1 clone evolution could be an indicator for TNBC prognosis (98).

### Dacinostat

Dacinostat (LAQ-824) has been observed to tackle cancer chemoresistance in multiple myeloma ad acute myeloid leukemia (99). One study demonstrated that dacinostat and givinostat can restore the activity of cytotoxic T lymphocytes in in pancreatic cancer cells (100). NVP-LAQ824 attenuated tumor growth and angiogenesis and enhanced VEGFR inhibitor PTK787/ZK222584-mediated inhibition of angiogenesis *via* upregulation of p21 and downregulation of angioprotein-2, Tie-2, VEGF, HIF-1α, and survivin (101). Using an orthotopic breast tumor model, NVP-LAQ824 plus PTK787/ZK222584 induced a greater suppression of tumor growth (101). LAQ824 can regulate the expression of miRNAs in SKBR-3 breast cancer cells (102). It has been known that noncoding RNAs, including microRNAs, lncRNAs and circRNAs, are critical in carcinogenesis in a variety of human cancers (103–105). LAQ824 increased 22 miRNA expressions and decreased 5 miRNA expressions in breast cancer cells (102). LAQ824 in combination with 5-Aza-2’-deoxycytidine, known as decitabine, displayed a greater antineoplastic effect on breast cancer cells (106). LAQ824 reduced the expression of ERα, PRβ, c-Myc, cyclin D1 and HDAC6 in breast cancer cells, leading to suppression of cellular proliferation (107). LAQ-824 sensitized drug sensitivity, including taxotere, epothilone B, trastuzumab and gemcitabine, *via* downregulation of HER-2 expression in breast cancer cells (108). LAQ824 was found to work as a sensitizer to immunotherapy with adoptive T-cell transfer in melanoma (109). Further exploration is pivotal to determine the LAQ824-enhanced immunotherapy in cancer patients *via* improving the anticancer function of tumor antigen-specific lymphocytes.

### Other HDACs

Pracinostat (SB939) attenuated tumor growth and metastasis *via* blocking the IL6/STAT3 pathway in breast cancer (110). YF479, a HDACi, exhibited antitumor functions in breast cancer, including suppression of growth, metastasis and recurrence (111). NK-HDAC-1 was designed and synthesized for fighting breast cancer, which induced apoptosis and cell cycle arrest *via* upregulation of p21 and inhibition of Cyclin D1 (112). Givinostat (ITF2357) increased cell death and reduced cell proliferation in urothelial carcinoma cells and acute lymphocytic leukemia (113, 114). Givinostat enhanced CTL sensitivity in pancreatic cancer cells (100). In addition, givinostat reduced cancer stemness and reversed transformed phenotype in glioblastoma (115, 116). The function of givinostat is breast tumorigenesis is unclear, which should be explored in the future. Tubastatin A and alisertib reduced the number of pulmonary metastases *via* suppression of HDAC6 and AURKA in breast tumor xenograft models (117). Tubastatin A in combination with palladium nanoparticles triggered cell apoptosis in breast cancer cells (118). MPT0G211, a HDAC6 inhibitor, exhibited an inhibition of tumor metastasis *via* attenuation of HDAC6 activity in breast cancer cells (119).

Trichostatin A (TSA) inhibited the expression of DNMT1 (DNA methyltransferase 1) *via* reduction of DNMT1 mRNA stability in Jurkat T leukemia cells (120). TSA decreased the transcript and protein levels of aromatase CYP19 and phospholipase C gamma-1 (PLC-γ1) in MCF-7 breast cancer cells (121, 122). SK-7041, a HDACi *via* a hybrid of TSA and MS-275, induced cell apoptosis and G2/M arrest in breast cancer cells (123). MAGE-C1 (melanoma-associated antigen-C1) and MAGE-C2 expressions were linked to advanced tumor grade and poor survival in breast cancer patients. TSA treatment increased 5-aza-CdR-induced MAGE-C2 transcription in breast cancer cells, indicating that MAGE-C2 could be a target for cancer immunotherapy (124). Tucidinostat, an inhibitor of HDAC1, HDAC2, HDAC3 and HDAC10, has shown a remarkable anticancer activity and a synergistic ability with immunotherapy (125). Tucidinostat combined with selinexor, an exportin 1 inhibitor, showed a greater antitumor effect on TP53 wild-type breast cancer (126). Breast cancer patients with HR+/HER2- received CDK4/6 inhibitor treatment and then obtained tucidinostat-based therapy, which displayed better clinical outcomes (127). DNMT inhibitor 5-zazcytidine and HDACi butyrate ameliorated the tumorigenicity of CSCs and retarded breast tumor growth (128). We believe more HDAC inhibitors will be developed for potentiating immunotherapy in the future.

## Conclusion and perspectives

**Figure 1:**
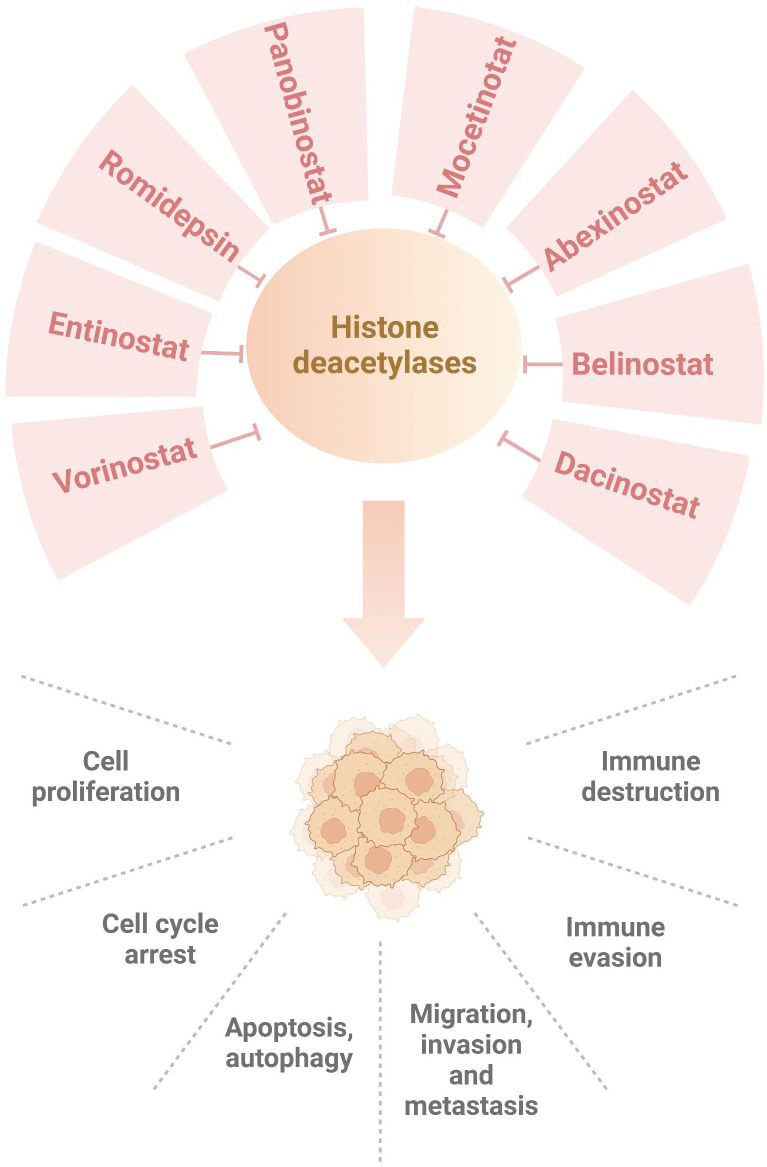
Numerous HDAC inhibitors suppress histone deacetylases in breast cancer. Dacinostat, belinostat, abexinostat, mocetinotat, panobinostat, romidepsin, entinostat and vorinostat, inhibit histone deacetylases and regulate breast tumorigenesis, progression and immunotherapy.

In conclusion, HDAC inhibitors improve immunotherapy *via* targeting HDACs and their downstream targets in breast cancer (**Figure 1**). Although HDAC inhibitors might be useful to enhance tumor immunotherapy, several concerns should be mentioned. So far, only five HDAC inhibitors have been approved by FDA for cancer therapy, including vorinostat, belinostat, panobinostat, pracinostat and romidepsin (129). These HDAC inhibitors exhibited clinical advantage in hematological malignancies. It is required to measure the efficacy of HDAC inhibitors in solid tumors (130). Sirtuins inhibitors, such as nicotinamide, sirtinol and splitomicin, have shown their activities in regulation of metabolism, DNA repair, proliferation, drug resistance and immunotherapy (131). Due to limited space, we do not discuss the role of sirtuins inhibitors in modulation of breast cancer immunotherapy. Among dozens of HDAC inhibitors, which one is the best choice for enhancement of immunotherapy in breast cancer? The development of inhibitors based on the differential expression of HDAC isoforms is pivotal to rationally develop selective and effective inhibitors for personalized-medicine treatment (132, 133). Notably, HDAC inhibitors also have adverse side effects and cause drug resistance, which should be overcome. The resistant reasons of HDAC inhibitors are still incomplete. This might be due to cancer cell types, tumor-specific mutations, tumor microenvironmental conditions, upregulation of efflux pumps (P-glycoprotein), overexpression of HDAC enzymes. Lastly, triple combination of HDACi, immunotherapy and other inhibitors could be a promising approach for the treatment of breast cancer.

## Author contributions

BL wrote the manuscript and made the figure. XC and KS edited and revised the manuscript. All authors contributed to the article and approved the submitted version.

## Glossary

**Table:**

ATF	activating transcription factor
BET	bromodomain and extra C-terminal
Bim1	a BH3 domain-containing pro-apoptotic protein
CDK	cyclin-dependent kinase
CHOP	CAAT/enhancer binding protein homologous protein
eIF2	eukaryotic translation initiation factor
ER	estrogen receptor
ERK1/2	extracellular signal-regulated kinase 1/2
HER2	human epidermal growth factor receptor 2
HIF-1	hypoxia-inducible factor-1
HLA	human leukocyte antigen
HTLV-1	human T-lymphotropic virus type 1
IFN-g	interferon gamma
LAG3	lymphocyte activation gene-3
MAPK	mitogen-activated protein kinase
MDSC	myeloid-derived suppressor cells
MHC	major histocompatibility
MMP	matrix metalloproteinase
mTOR	mammalian target of rapamycin
NF-kB	nuclear factor-kappa B
NLR	neutrophil-to-lymphocyte ratio
PARP	poly ADP-ribose polymerase
PDX	patient-derived xenograft
PI3K	phosphatidyl inositol 3 kinase
PTEN	phosphatase and tensin homolog
PR	progesterone receptor
ROS	reactive oxygen species
STAT	signal transducers protein kinase
TIM-3	T-cell immunoglobulin domain and Mucin domain 3
TIMP-1	tissue inhibitor of metalloproteinase-1
TNBC	triple-negative breast cancer
TRAIL	tumor necrosis factor-related apoptosis-inducing ligand
Treg	T-regulatory cell
UPR	unfolded protein response
VEGF	vascular endothelial growth factor.
